# Milk Supplemented with Organic Iron Improves Performance, Blood Hematology, Iron Metabolism Parameters, Biochemical and Immunological Parameters in Suckling Dalagh Lambs

**DOI:** 10.3390/ani12040510

**Published:** 2022-02-18

**Authors:** Mohammad Asadi, Abdolhakim Toghdory, Maryam Hatami, Jalil Ghassemi Nejad

**Affiliations:** 1 Department of Animal and Poultry Nutrition, Animal Science Faculty, Gorgan University of Agricultural Sciences and Natural Resources, Gorgan 49189-43464, Iran; mohammadasadiseyed1994@yahoo.com (M.A.); toghdory@yahoo.com (A.T.); 2 Department of Animal Science, Faculty of Agriculture, University of Tabriz, Tabriz 51666-16471, Iran; maryam.hatami928@gmail.com; 3 Department of Animal Science and Technology, Konkuk University, Seoul 05029, Korea

**Keywords:** antioxidants, hematology, organic iron, suckling lambs, thyroid hormones, weight gain

## Abstract

**Simple Summary:**

It is known that in early life, newborn lambs are prone to iron deficiency due to the type of placenta and low iron in milk. Hence, the influence of milk supplementation with iron in newborn lambs needs to be investigated. The objective of this study was to elucidate the effect of milk supplemented with organic iron on performance, blood hematology, iron metabolism parameters, together with biochemical and immunological parameters in suckling lambs. Our findings revealed that supplementation of milk with 25 mg/d organic iron could be used effectively as a feed supplement for improving weight gain, promoting health, as well as improving the oxidative status of suckling lambs retained at the plasma level.

**Abstract:**

This study was conducted to investigate the effect of milk supplemented with organic iron on performance, blood hematology, iron metabolism parameters, biochemical and immunological parameters in suckling lambs. Thirty-six newborn Dalagh lambs were randomly divided into three groups with 12 replications. The control group was fed with milk without organic iron. The other two groups were fed milk supplemented with 25 and 50 mg/d organic iron, respectively. During the experiment, increased daily weight gain and total body weight were observed in the iron-supplemented groups. An increase in the levels of red blood cell, hemoglobin, hematocrit, mean corpuscular hemoglobin, and mean corpuscular concentration in iron supplemented groups was indicated. Consumption of organic iron caused a significant decrease in plasma copper concentration. Total antioxidant status level was lower, but levels of glutathione peroxidase, superoxide dismutase, and catalase were higher in iron supplemented groups. In organic iron supplemented groups, insulin and thyroid hormones levels were significantly increased, and glucose level was significantly decreased. In organic iron supplemented groups, alkaline phosphatase level significantly increased, and aminotransferase level significantly decreased. Overall, the use of organic iron in the milk improved the performance and health in suckling lambs, and since a lower level of supplementation is naturally preferable, supplementation of milk with 25 mg/d organic iron is recommended.

## 1. Introduction

Iron is a necessary trace element for the preservation of animal health and efficiency [1,2]. Iron plays a pivotal role in several metabolic processes and is essential for synthesis of DNA, RNA and proteins in the body. Iron is necessary for the synthases of cellular enzymes including oxidases, catalase peroxidases, cytochromes, ribonucleotide reductases, aconitases, and nitric oxide [3]. Supplementation of diet with iron increased hematological parameters and improved lamb growth [4]. In addition, the presence of iron in the diet was found to be effective in increasing appetite, secretion of thyroid hormones, and glucose metabolism [4]. It has been well established that changes in iron intake can influence copper metabolism in animals and consumption of iron reduced the plasma copper concentration via its antagonistic relationship with copper uptake [5]. Consumption of iron in the diet of newborn lambs reduced the concentration of calcium and phosphorus in plasma [6]. Furthermore, additional iron intake in calves increased the number of red blood cells and hemoglobin concentrations [7].

On the other hand, iron deficiency is the most common nutrient deficiency throughout the world and causes metabolic disorders that adversely affect health conditions [8]. Iron deficiency seems to affect the antioxidant defense system [9]. The malfunction of an antioxidant system could in turn affect the metabolism of minerals such as copper and selenium. A recent study demonstrated that iron deficiency without anemia is associated with decreased levels of blood copper and ceruloplasmin and reduced superoxide dismutase activity in erythrocytes [10] while iron deficiency has been associated with biomarkers of oxidative stress [11]. Furthermore, high levels of iron in the body can induce peroxidative injury via production of reactive oxygen species including loss of functional integrity and decreased turnover of epithelial cells, as also with marked mucosal cell death [12]. Distribution of iron supplements in young animals, food fortification, and diversification of diets are the basic methods to reduce iron deficiency in animals [13]. The anemia due to iron deficiency (iron deficiency anemia, IDA) is the most prevalent and widespread nutritional disturbance in the world [13].

Animals’ need for iron varies according to age, sex, and body condition [14,15]. The dietary iron requirement for sheep was set at 30 mg Fe/kg dry matter (DM) by the National Research Council [16]. Young animals in the early steps of life are the most vulnerable to iron deficiency because animal production is affected by events from the primary phase of life, when still in the mother’s womb. Insufficient transmission of required minerals from the mother to the fetus can result in deficiency of nutrients for the offspring and disorders in metabolism [17]. Despite a lamb’s survival, the associated intrauterine growth restriction (IUGR) would cause increased sensitivity to disorders in the nerve, respiration, intestine, and circulation [18]. On the other hand, iron deficiency in the milk, because the access to soil as the main source of dietary iron for farm animals is limited [19], may result in the development of anemia in suckling lambs.

At the present time, iron supplementation is based primarily on inorganic compounds. However, these compounds can undergo oxidation and be transformed into insoluble forms [20]. Several previous reports confirm increased bioavailability of trace elements when animals’ feed was supplemented with metal chelates (e.g., with amino acids and peptides), resulting in an improvement of growth and general health condition compared with the deficiency amounts to meet the animals’ needs [21,22]. At present, such types of metal complexes are available commercially to be used in animal feeding. [23].

Since there is no mechanism for iron excretion, toxicity depends on the level of iron already existing in the body. As a result, some animals develop clinical signs of toxicosis even when they receive doses that cause no problems in other animals. Intravenous injection of iron potentially causes the most toxicity. Intramuscular injections are less toxic and produce the least benefit when supplemented in the diet. This is probably because the amount of iron absorbed orally is not 100% of the dose [24]. It is characterized primarily by gastrointestinal effects, for example vomiting, diarrhea, gastrointestinal bleeding, lethargy, a recurrence of gastrointestinal signs, metabolic acidosis, shock, hypotension, tachycardia, cardiovascular collapse, coagulation deficits, hepatic necrosis, and possibly death [24].

Therefore, the objective of this study was to test the effects of organic iron supplementation in the milk on performance, blood hematology, iron metabolism parameters, as well as biochemical and immunological parameters in suckling lambs.

## 2. Materials and Methods

### 2.1. Experimental Design, Animals, and Treatments

This research was conducted in the research station of the Department of Animal Sciences, Gorgan University, Iran. A total 36 newborn Dalaq male lambs were used. Following parturition, the navel of each lamb was disinfected by povidone iodine, and the lambs were weighed and transferred to individual pens. Lambs were housed in individual boxes of 1 × 1 m with a concrete floor covered with chaff. The initial average BW of each group is reported in Table 1. Within the initial hour of life, 10% of body weight of pooled herd colostrum was fed by nipple bottle. Colostrum feeding was continued for the second day of life. At 2 days of age, lambs were divided into 3 treatments with 12 replications. Lambs were fed with herd milk supplemented with organic iron for up to 30 days of life (10% of body weight, twice daily). The organic iron supplementation in the milk was completely dissolved. Water was provided ad libitum in the buckets in each pen individually. Experimental treatments were as follows: T1 (control group): with milk without organic iron supplementation; T2: fed with 25 mg/d organic iron supplementation; T3: fed with 50 mg/d organic iron supplementation. The organic iron (Amino Fe) used in this study consisted of an iron element that is ionically bound to organic molecules (peptides and amino acids). To examine weight changes, the lambs were weighed on days 10, 20, and 30.

### 2.2. Blood Samples, and Laboratory Analyses

To measure hematological, biochemical and immunological parameters, blood samples were obtained from the jugular vein of lambs on day 30. Then, blood samples were transferred into a tube containing K2EDTA (anticoagulant) (Sarstedt, Warsaw, Poland). To prepare the plasma, blood samples were centrifuged at 3000× *g* for 10 min at room temperature. Then, the plasma samples were frozen (−20 °C) until the analysis.

Hematological parameters including red blood cells (RBC), hemoglobin (HGB), hematocrit (HCT), platelet count test (PLT), mean cell volume (MCV), mean corpuscular hemoglobin (MCH), mean corpuscular hemoglobin concentration (MCHC), white blood cells (WBC), neutrophils (NEUT), eosinophils (EOS), lymphocytes (LYMPH), and monocytes (MONO) were measured using an automatic cell count (Automatic Syfmex model NKX-21) [25].

Concentrations of iron, unsaturated iron binding capacity (UIBC), total iron binding capacity (TIBC), transferrin saturation, transferrin, total antioxidant status (TAS), calcium, phosphorus, zinc, copper, glutathione peroxidase (GPx), superoxide dismutase (SOD), catalase (CAT), malondialdehyde (MDA), glucose and activities of aminotransferase (AST), alkaline phosphatase (ALP), alanine aminotransferase (ALT), creatine phosphokinase (CPK), and lactate dehydrogenase (LDH) enzymes were measured using kits manufactured by Pars Azmoun Company (Pars Azmoun, Iran), using a photometric spectrometer (UV-Vis model 365 LAMBDA, Perkinelmer, NY, USA) with the emission wavelength specific for each element.

Immunological parameters including tumor necrosis factor (TNF-α), immunoglobulin G (IgG), immunoglobulin M (IgM), as well as concentrations of insulin, insulin-like growth factor (IGF-1), and thyroid hormones including triiodothyronine (T3) and tetraiodothyronine (T4) were determined in plasma by the Pars Azmon Company kit (Pars Azmoun, Iran) by ELISA (ELX808. TEX-Bio, BioTek Instruments, Frankfurt, Germany).

### 2.3. Statistical Analysis

Data were analyzed as a completely randomized design arrangement of 3 treatments and 12 replicates using the GLM procedure of SAS (version 9.1, SAS Institute Inc., SAS Campus Drive, Cary, NC, USA). A comparison of means by Duncan’s multiple range tests was carried out at the probability 5% level.

## 3. Results and Discussion

### 3.1. Performance

The results of the effect of organic iron supplementation on weight gain are reported in Table 1. There were significant differences for daily weight gain and total body weight between the experimental groups (*p* < 0.05). During the whole period and from 1 to 10, 11 to 21, and 21 to 30 days of age, higher daily weight gain and total body weight were observed in the iron-supplemented groups compared to the control group (*p* < 0.05). Milk offered in iron-supplemented groups was higher than those of the control group, but there was no significant difference (*p* > 0.05).

Minerals play an important role in the initial stages in life of both humans and animals [26]. Iron-amino acid chelate was used for food fortification [27] as well as animal supplements [28]. In agreement with our results, Eisa and Elgebaly [29] indicated that iron administration significantly increased the total BW and daily weight gain in calves. Mohri et al. [30] found improved weight gain in calves by the administration of iron. Consistent, improved total and average daily gains in neonatal dairy calves by oral administration of iron at a dose rate of 150 mg/d for 28 days were observed [31]. Additionally, the weight of the animals including total, weekly, and daily gains were increased after parenterally supplementation of iron dextran at a dose rate of 1000 mg/animal [32]. Given this, Mohri et al. [30] suggested that adequate iron for normal appetite, secretion of IGF-I and T3, and glucose uptake is pivotal.

In contrast to our results, Moosavian et al. [33] and Bostedt et al. [34] reported no significant effect of iron supplementation on the BW of newborn calves. Addition of ferrous sulfate to the diet for 135 days did not have a significant effect on the daily weight gain, total weight gain, and feed consumption of fattening lambs [35]. Hansen et al. [36], also reported that high levels of iron supplementation (750 mg of ferrous sulfate per kg of dietary dry matter) in growing calves reduced feed efficiency over a 56-day period.

The various effects of iron supplementation on lambs’ performance may be due to the different agents including iron storage, dosage, method of iron supplementation, and the level of dietary iron [37]. For instance, one of the effective factors in reducing feed consumption when using high levels of iron is due to reduced palatability of the diet [35]. The reason for the difference between the present results and the few aforementioned studies can be attributed to the type of livestock, type of iron supplement used, and breeding conditions.

### 3.2. Hematological Parameters

The results of the effect of organic iron supplementation on hematological parameters are reported in Table 2. The use of organic iron supplements significantly increased the levels of RBC, HGB, HCT, MCH, and MCHC in groups that received organic iron (*p* < 0.05) but, iron supplementation had no significant effect on the levels of PLT, MCV, WBC, NEUT, EOS, LYMPH, and MONO (*p* > 0.05).

In agreement with our results, it was indicated that administration of iron, orally or parenterally affected blood hematology in general [29,30,32]. Different studies have shown that administration of iron led to an increase in RBC parameters in calves [8,29,31,33,38,39]. A single dose of 500 mg iron dextran to suckling calves resulted in increased RBC and MCV within the first month of life leading to hampered preweaning nutritional anemia [38]. Nonetheless, finding differences in hematological parameters after weaning could be partially explained by the ingested food supply [38]. In agreement with the results of this study, injection of 500 mg iron (as polymaltose hydroxide) to newborn dairy calves resulted in increased RBC. This phenomenon indicates that the iron requirements of newborn calves could be more than the recommended level [8]. Kupczynski et al. [8] showed that iron supplementation to suckling calves could decrease MCV and increase MCHC. Injection of 500 mg Fe per calf at 48–24 h of age had a significant effect on hematological parameters and increased RBC, hemoglobin (HB), and hematocrit (HTC) in calves [8].

A previous study revealed the role of oral supplementation of iron (100 mg/d) significantly caused prevention of anemia in calves at both clinical and sub-clinical level [40]. It has been reported that Fe-dextran administration (1000 mg and 1500 mg) resulted in a permanent peripheral iron level and increased HB level in the early development of calves [34]. The effectiveness was observed over the 6 weeks of the study, however, the control group showed a decrease in peripheral iron and HB levels over the same period.

In agreement with our study, Kupczynski et al. [8] reported that feeding protein-iron complex had no significant effect on WBC in preweaning calves. Norouzian et al. [41] indicated that parenteral administration of cobalt, copper, and iron had no significant effect in late pregnancy on Neut, EOS, Lymph, and Mono in ewe. Parenteral administration of iron and copper significantly reduced the note level in neonatal calves, but had no effect on Lymph and Mono [32]. Parenteral over-supplementation of vitamin A and iron did not increase the levels of WBC, NOTE, and LYMPH in neonatal dairy calves [33].

The iron is associated with metabolic and hematological parameters [42]. Adequate iron content in the feed is essential for the manufacture of RBC and HB. A progressive reduction in amounts of HCT, RBC, and HB occurs over the early weeks of life [8,43,44]. Deficiencies occur most likely in young animals because of low iron in milk [32]. Red blood cell parameters are widely used to monitor erythropoiesis, and adequate erythropoiesis is necessary for sufficient iron supply [29]. Hemoglobin is made up of a protein called globin and an iron pigment called heme. Iron is needed for heme and therefore, in the case of iron deficiency, the synthesis of hemoglobin is reduced [4]. Most studies have focused on hematology of newborn lambs and calves but other studies showed that administration of iron caused an increase in RBC parameters and MCV in calves [32,34,45].

### 3.3. Iron Metabolism Parameters, and Concentration of Minerals

The results of the effect of organic iron supplementation on iron metabolism parameters and other minerals are reported in Table 3. Levels of iron, transferrin saturation, and transferrin showed significant changes among the experimental groups, and were higher in organic iron supplemented groups (*p* < 0.05). However, the levels of UIBC and TIBC showed no significant difference between the experimental groups (*p* > 0.05).

Organic iron supplementation in suckling lambs showed a significant decrease in plasma copper concentration (*p* < 0.05). However, concentrations of calcium, phosphorus, and zinc were not affected by organic iron supplementation in suckling lambs (*p* > 0.05).

In agreement with our results, Heidarpour Bami et al. [32] reported a numerical increase in serum iron levels in iron-receiving neonatal dairy calves. However, there was no significant difference in UIBC and TIBC between the experimental groups in this study. In contrast to our results, Mohri et al. [45] showed levels of TIBC were significantly higher in calves receiving more iron doses (150 mg/d) in comparison to the control group, and on the 28th day significantly lower. Iron deficiency initially is due to a decrease of the iron in serum and an increase of the total iron binding capacity (TIBC) along with the unsaturated iron binding capacity (UIBC) [46].

Depending on rearing management, Reece and Hotchkiss [47] suggested that the utilization of milk replacer could result in higher iron levels in calves and saturation of transferrin leading to lower TIBC [47]. Iron deficiency is associated with higher iron binding capacity, whereas significantly lower levels of TIBC have been observed [32]. Administration of iron did not affect the TIBC and transferrin saturation; however, the transferrin level was significantly increased in the trial groups. The highest of the transferrin levels were observed in neonatal calves at the 28th day [8]. Excess iron in the body is bound to transferrin [48]. The difference between the results of our study and the others could be attributed to the use of different forms of iron supplementation [8].

There are close relationships between the metabolisms of various trace elements, including iron, based on antagonistic or synergistic interactions [49]. Higher content of trace elements in contrast to iron, such as cobalt, zinc, copper, chrome, and calcium, which impair iron uptake or its physiological effect, can impair the metabolism of this element, and vice versa [49]. Various studies have reported that iron administration increases serum iron concentrations in ruminants [50]. Injection of iron into suckling calves caused a significant increase in serum iron concentration [33]. Moreover, administration of iron-dextran in periods of anemia showed a significant increase in serum iron concentration [51].

On the other hand, administration of iron in the form of carbonate iron in heifers’ diets for 32 weeks led to a decrease in plasma copper concentration [52]. In addition, iron supplementation in the form of ferrous sulfate in the diet of rams reduced the plasma copper concentration compared to the control group [53]. Increasing iron supplementation in the diet due to competition with copper and its antagonistic relationship during absorption from the intestine reduced the plasma copper concentration [5].

Contrary to the present results, Heidarpour Bami et al. [32] showed that injecting dextran iron in Holstein heifers had no effect on iron and copper concentrations in the blood of suckling calves. In addition, injection of dextran iron into newborn calves did not cause significant changes in serum iron concentration among experimental groups. This may be due to the interval of 12 to 13 days between iron injection and the first measurement of serum concentration [19].

Consistent with the results of the present study, Ali Arabi et al. [54] did not observe any difference in plasma zinc concentrations among the experimental groups. In another study, Prabowo et al. [55] reported that iron supplementation in the diets of growing lambs had no effect on plasma zinc concentrations. According to the results of Ali Arabi et al. [54] plasma calcium and phosphorus concentrations were the same in all groups and the difference between treatment groups was not significant. Consumption of iron in the diet of newborn lambs reduced the concentration of calcium and phosphorus in their plasma [6]. It has been shown that the absorption and transport of bivalent minerals such as iron, zinc, and copper in the small intestine of an animal is performed by transporters such as divalent metal transporter 1 (DMT1), so there is competition between these elements during uptake [56]. For this reason, it is necessary to measure the concentrations of these elements when administering iron. On the other hand, the amount of iron absorption varies with the type of feed. In addition to the degree of saturation of living organisms with this nutrient, the type of feed consumed, the physical and chemical shape of iron, and the interaction between the components of the diet are also important [57]. Taken together, the differences in the results of various studies can be due to the form of iron consumption and the type of animal. Since there is little information about the effect of iron on mineral metabolism in suckling lambs, more research is needed to investigate its effects in suckling lambs.

### 3.4. Antioxidation Status, Biochemical and Immunological Parameters

The results of the effect of organic iron supplementation on antioxidation status, biochemical and immunological parameters are reported in Table 4. The level of TAS was lower in organic iron supplemented groups compared to the control (*p* < 0.05); while the levels of GPx, SOD, and CAT were higher in organic iron supplemented groups (*p* < 0.05). In organic iron supplemented groups, the level of insulin significantly increased, and the level of glucose significantly decreased (*p* < 0.05). Additionally, a significant increase in thyroid hormones was observed in organic iron supplemented groups (*p* < 0.05). In organic iron supplemented groups, the level of ALP was increased significantly, and the level of AST decreased (*p* < 0.05). The levels of IGF-1, TNF-α, IgG, IgM, ALT, CPK, LDH, and MDA showed no change among the experimental groups (*p* > 0.05).

The total antioxidant assay contains all the biological ingredients that help to assess the activity in the realm of preventing extreme oxidation [58]. In other words, assessment of the main antioxidant enzymes that incudes SOD and GPx helps to aim the assessment of the activity of intracellular antioxidants [59]. In addition, iron is one of the most essential parts of the catalase enzyme (which is present in RBCs) and as an antioxidant, somatic cells are preserved with high levels of hydrogen peroxide by converting it to oxygen [60].

In agreement with the results of the present study, administration of high levels of iron led to higher SOD activity in newborn calves [8]. Extreme levels of iron in rats caused a significant increase in CAT and SOD activities [61]. Zhang et al. [62] indicated that antioxidant enzymes including TAS and GPx activity were significantly increased following 10 g/d administration of iron in newborn calves. Fernández-Real et al. [63] showed that supplementation of iron increased antioxidant activity and decreased oxidative factors in iron deficient patients. Kupczynski et al. [8] indicated TAS levels were significantly different between iron receiving groups [8] and in the final day of iron administering, lower TAS was observed in the serum of the experimental calves, that may be explained by a lower supply of free radicals [8]. In contrast to our results, no differences were shown between iron receiving groups in serum insulin and glucose levels; however, when the higher levels of iron were supplemented, lower mean glucose and higher insulin levels were observed [8]. Differences in glucose absorption in animals are likely due to the various breeding conditions. With milk replacer feed versus colostrum or raw milk, inadequate glucose absorption was indicated in neonatal calves [64].

IGF-1 is a pleiotropic hormone exerting mitogenic and anti-apoptotic effects [65]. Knowing that IGF-1 is mainly produced by the liver, it can be obtained via the preparation of energy feeds, nutrients, minerals, and vitamins, and by the effects of non-nutritive agents [59,66] Studies on animal models and humans have shown a meaningful relationship between iron and IGF-1 [67,68]. Prolonged iron deficiency resulted in decreased blood HB, following a decreased IGF-1 secretion by the liver [68].

In agreement with our results, levels of IGF-1 were unaffected by iron supplementation [33]. In addition, the level of feeding was associated with the level of IGF-1 in the blood. The IGF-1 levels in the blood depend on the level of nutrition. Feeding diets containing high protein and energy to the calves resulted in a higher plasma level of IGF-1 than those fed with moderate or low protein and energy diets [69]. Iron supplementation to young dairy cattle long-term resulted in increased IgG and IGF-1 levels [70]. Thyroid hormone and IGF-I levels dropped in calves born prematurely [71]. Consistent with our results, the levels of IgG or IgM in serum were unaffected by the diet and time of blood sampling [8]. Infections can stimulate immune response, with simultaneous production of cytokines such as TNF-α [72]. Iron administration had no effect on TNF-α level in the experimental groups [8] as a similar pattern was shown in our results.

In agreement with our results, higher levels of T3 and T4 were observed by oral supplementation of iron to neonatal calves in comparison with the control [29]. Ceppi and Blum [73] indicated that the levels of T3 were significantly influenced by iron consumption in calves, but there was no effect on the levels of T4. In another study, no difference was observed between experimental groups with respect to thyroid metabolism [37].

Stojić et al. [74] suggested that a high level of readily available stores of thyroid hormones should be frequently used for adapting to the new environmental condition particularly in the initial critical days of life. Presence of some trace elements such as iron, iodine, selenium, and zinc are required for synthesis and metabolism of thyroid hormones. Any deficiencies in these elements can destroy thyroid actions [75]. In precedent studies, thyroid hormone metabolism disorders were indicated due to iron deficiency [76,77]. The role of iron in thyroid metabolism is less known. One reason is that thyroid hormones impairment in iron deficiency status is apparently due to a decrease in the activity of the enzyme thyroid peroxidase (an iron-dependent enzyme); this enzyme is involved in the synthesis of thyroid hormones [78]. It has been found that deficiency or poisoning with this element has a negative effect on the metabolism of thyroid hormones [79].

Stability and integrity of hepatocyte membranes are essential for vital liver function [80]. Alanine aminotransferase (ALT) is a specific enzyme for the liver and any damage to liver cells increases the release of this enzyme [81]. Additionally, bile duct obstruction increased the serum concentration of ALP enzyme [81]. Increased concentrations of AST and ALT enzymes may be due to increase of anabolism or decreased catabolism [81]. Therefore, in the present study, due to bile duct obstruction and dysfunction under the influence of organic iron supplementation, the amount of AST and ALP enzymes was affected. Further research is needed to investigate the role of iron on liver enzymes in ruminants.

## 4. Conclusions

The results indicate that organic iron could be used as a feed supplement in the milk for improving weight gain, promoting health, as well as for improving the oxidative status of suckling lambs at the plasma level. Therefore, in this study, the use of 25 mg/d organic iron supplement in suckling lambs is recommended since both levels of 25 and 50 mg/d produced a similar effect.

## Figures and Tables

**Table 1:** The effect of organic iron supplementation on suckling lambs.

Item	Treatment ^1^			SEM	*p*-Value
	Control	25 mg/d	50 mg/d		
Initial weight (kg)	4.33	4.28	4.40	0.148	0.1339
Daily gain (1–10 d, g/d)	202.56 ^b^	234.97 ^a^	238.85 ^a^	11.981	0.0319
Body weight (10 d, kg)	6.36 ^b^	6.63 ^a^	6.79 ^a^	0.249	0.0307
Daily gain (11–20 d, g/d)	191.82 ^b^	220.88 ^a^	228.26 ^a^	16.308	0.0118
Body weight (20 d, kg)	8.28 ^b^	8.84 ^a^	9.07 ^a^	0.447	0.0289
Daily gain (21–30 d, g/d)	168.57 ^b^	194.92 ^a^	190.85 ^a^	11.607	0.0074
Body weight (30 d, kg)	9.97 ^b^	10.79 ^a^	10.98 ^a^	0.675	0.0048
Total daily gain (g/d)	188.12 ^b^	217.09 ^a^	219.33 ^a^	8.179	0.0001
Total body weight (kg)	5.64 ^b^	6.51 ^a^	6.58 ^a^	0.248	0.0001
Milk offered (1–30 d, g/d)	639.80	657.02	670.33	32.147	0.0560

^a,b^ Within a row, means without a common superscript differ significantly (*p* < 0.05). ^1^ Treatment = T1) milk without organic iron supplementation (control); T2) 25 mg/d organic iron supplementation, T3) 50 mg/d organic iron supplementation.

**Table 2:** The effect of organic iron supplementation on hematological parameters.

Item	Treatment ^1^			SEM	*p*-Value
	Control	25 mg/d	50 mg/d		
RBC ^2^, T/L	8.80 ^b^	13.04 ^a^	12.95 ^a^	1.158	0.0015
HGB ^3^, mmol/L	6.49 ^b^	8.90 ^a^	9.12 ^a^	0.732	0.0271
HCT ^4^, L/L	0.24 ^b^	0.42 ^a^	0.45 ^a^	0.071	0.0148
PLT ^5^, G/L	841.28	819.11	824.70	42.09	0.2147
MCV ^6^, fl	29.51	31.29	31.02	2.087	0.2002
MCH ^7^, fmol	0.75 ^b^	0.84 ^a^	0.82 ^a^	0.251	0.0224
MCHC ^8^, mmol/L	18.58 ^b^	22.75 ^a^	23.12 ^a^	0.847	0.0225
WBC ^9^, G/L	9.00	8.75	8.66	0.738	0.5958
NEUT ^10^, %	9.20	9.50	9.25	0.278	0.6788
EOS ^11^, %	34.37	32.91	34.12	6.659	0.9803
LYMPH ^12^, %	2.26	2.29	2.29	0.062	0.3606
MONO ^13^, %	55.27	55.56	54.47	4.157	0.5773

^a,b^ Within a row, means without a common superscript differ significantly (*p* < 0.05). ^1^ Treatment, T1) milk without organic iron supplementation (control); T2) 25 mg/d organic iron supplementation, T3) 50 mg/d organic iron supplementation. ^2^ RBC, red blood cells. ^3^ HGB. hemoglobin. ^4^ HCT, hematocrit. ^5^ PLT, platelet count test. ^6^ MCV, mean cell volume. ^7^ MCH, mean corpuscular hemoglobin. ^8^ MCHC, mean corpuscular hemoglobin concentration. ^9^ WBC, white blood cells. ^10^ NEUT, neutrophils. ^11^ EOS, eosinophils. ^12^ LYMPH, lymphocyte. ^13^ MONO, monocytes.

**Table 3:** The effect of organic iron supplementation on iron metabolism parameters and other minerals.

Item	Treatment ^1^			SEM	*p*-Value
	Control	25 mg/d	50 mg/d		
Iron, mol/L	14.33 ^b^	16.28 ^a^	17.40 ^a^	0.748	0.0339
UIBC ^2^, mol/L	5.97	6.56	6.85	0.481	0.0549
TIBC ^3^, mol/L	18.41	18.00	17.78	0.554	0.0570
Transferrin saturation, %	69.82 ^b^	78.88 ^a^	79.26 ^a^	1.308	0.0018
Transferrin, mg/mL	2.98 ^b^	4.87 ^a^	5.01 ^a^	0.421	0.0001
Calcium, ng/dL	96.01	95.85	94.97	0.178	0.4128
Zinc, ng/dL	1.46	1.35	1.39	0.032	0.2100
Phosphorus, ng/dL	61.82	61.36	62.01	0.229	0.3485
Copper, ng/dL	0.81 ^a^	0.62 ^b^	0.59 ^b^	0.017	0.0001

^a,b^ Within a row, means without a common superscript differ significantly (*p* < 0.05). ^1^ Treatment, T1) milk without organic iron supplementation (control); T2) 25 mg/d organic iron supplementation, T3) 50 mg/d organic iron supplementation. ^2^ UIBC, unsaturated iron binding capacity. ^3^ TIBC, total iron binding capacity.

**Table 4:** The effect of organic iron supplementation on antioxidation status, biochemical and immunological parameters.

Item	Treatment ^1^			SEM	*p*-Value
	Control	25 mg/d	50 mg/d		
TAS ^2^, mmol/L	1.13 ^a^	0.92 ^b^	0.90 ^b^	0.148	0.0001
GPx ^3^, nmol NADPH ox/mg	5.64 ^b^	7.51 ^a^	7.58 ^a^	0.248	0.0001
SOD ^4^, U/mg of proteins	18.74 ^b^	31.24 ^a^	33.97 ^a^	2.104	0.0001
CAT ^5^, U/mg of proteins	0.49 ^b^	0.58 ^a^	0.62 ^a^	0.010	0.0001
MDA ^6^, nmol/mL	1.24	1.17	1.14	0.047	0.2356
Insulin, ng/mL	0.56 ^b^	0.67 ^a^	0.65 ^a^	0.081	0.0111
Glucose, mmol/L	5.96 ^a^	5.43 ^b^	5.49 ^b^	0.049	0.0217
IGF-1 ^7^, ng/mL	48.82	50.88	47.26	1.372	0.0618
TNF-α ^8^, pg/mL	102.28	99.84	99.07	1.407	0.0689
IgG ^9^, mg/mL	13.30	12.56	12.98	0.498	0.5451
IgM ^10^, mg/mL	0.79	0.74	0.78	0.051	0.1874
T_3_^11^, nmol/L	9.42 ^b^	10.74 ^a^	10.91 ^a^	0.192	0.0048
T_4_^12^, nmol/L	100.16 ^b^	117.09 ^a^	120.39 ^a^	5.075	0.1241
AST ^13^, u/L	66.14 ^a^	54.12 ^b^	52.29 ^b^	1.487	0.0314
ALT ^14^, u/L	16.08	15.86	15.45	0.670	0.1121
ALP ^15^, u/L	197.29 ^b^	227.46 ^a^	223.13 ^a^	9.241	0.0001
CPK ^16^, u/L	61.01	57.24	59.16	2.377	0.1684
LDH ^17^, u/L	241.01	223.74	238.99	19.755	0.3448

^a,b^ Within a row, means without a common superscript differ significantly (*p* < 0.05). ^1^ Treatment, T1) milk without organic iron supplementation (control); T2) 25 mg/d organic iron supplementation, T3) 50 mg/d organic iron supplementation. ^2^ TAS, total antioxidant status. ^3^ GPx, glutathione peroxidase. ^4^ SOD, superoxide dismutase. ^5^ CAT, catalase. ^6^ MDA, malondialdehyde. ^7^ IGF-1, insulin-like growth factor 1. ^8^ TNF-α, tumor necrosis factor. ^9^ IgG, immunoglobulin G. ^10^ IgM, immunoglobulin M. ^11^ T_3_, triiodothyronine. ^12^ T_4_, tetraiodothyronine. ^13^ AST, alkaline phosphatase. ^14^ ALT, alanine aminotransferase. ^15^ ALP, alkaline phosphatase. ^16^ CPK, creatine phosphokinase. ^17^ LDH, lactate dehydrogenase.

## Data Availability

Data are available upon a reasonable request.
